# Discovery of knock-down resistance in the major African malaria vector *Anopheles funestus*

**DOI:** 10.1111/mec.17542

**Published:** 2024-10-07

**Authors:** Joel O. Odero, Tristan P. W. Dennis, Brian Polo, Joachim Nwezeobi, Marilou Boddé, Sanjay C. Nagi, Anastasia Hernandez-Koutoucheva, Ismail H. Nambunga, Hamis Bwanary, Gustav Mkandawile, Nicodem J Govella, Emmanuel W. Kaindoa, Heather M. Ferguson, Eric Ochomo, Chris S. Clarkson, Alistair Miles, Mara K. N. Lawniczak, David Weetman, Francesco Baldini, Fredros O. Okumu

**Affiliations:** 1. Environmental Health and Ecological Sciences Department, Ifakara Health Institute, Ifakara, Tanzania; 2. School of Biodiversity, One Health, and Veterinary Medicine, G12 8QQ, University of Glasgow, Glasgow, UK; 3. Department of Vector Biology, Liverpool School of Tropical Medicine, L3 5QA, Liverpool, UK; 4. Entomology Section, Centre for Global Health Research, Kenya Medical Research Institute, Kisumu, Kenya; 5. Wellcome Sanger Institute, Wellcome Genome Campus, Hinxton, CB10 1SA, UK.

**Keywords:** Insecticide resistance, knock-down resistance (*kdr*), *Anopheles funestus*, Voltage-gated sodium channel (*Vgsc*), Tanzania

## Abstract

A major insecticide resistance mechanism in insect pests is knock-down resistance (*kdr*) caused by mutations in the voltage-gated sodium channel (*Vgsc*) gene. Despite being common in most malaria *Anopheles* vector species, *kdr* mutations have never been observed in *Anopheles funestus*, the principal malaria vector in Eastern and Southern Africa, with resistance mainly being conferred by detoxification enzymes. In a parallel study, we monitored 10 populations of *An. funestus* in Tanzania for insecticide resistance unexpectedly identified resistance to a banned insecticide, DDT, in the Morogoro region. Through whole-genome sequencing of 333 *An. funestus* samples from these populations, we found eight novel amino acid substitutions in the *Vgsc* gene, including the *kdr* variant, L976F (equivalent to L995F in *An. gambiae*), in tight linkage disequilibrium with another (P1842S). The mutants were found only at high frequency in one region and were accompanied by weak signatures of a selective sweep, with a significant decline between 2017 and 2023. Notably, *kdr* L976F was strongly associated with survivorship to exposure to DDT insecticide, while no clear association was noted with a pyrethroid insecticide (deltamethrin). The WHO prequalifies no DDT products for vector control, and the chemical is banned in Tanzania. Widespread DDT contamination and a legacy of extensive countrywide stockpiles may have selected for this mutation. Continued monitoring is necessary to understand the origin of *kdr* in *An. funestus*, and the threat posed to insecticide-based vector control in Africa.

## Introduction

Chemical insecticides are central to the control of agricultural pests and disease vectors, such as mosquitoes. The control of *Anopheles* mosquitoes through the distribution of over 2.9 billion insecticide-treated bed nets (ITNs) has helped avert an estimated 633 million cases of malaria (1), a disease that still kills 600,000 yearly (2). However, the widespread use of insecticides for agricultural pest and disease vector control also has detrimental consequences, including direct lethal and sub-lethal effects on human and animal health and destabilising effects on ecosystem structure and function (3, 4). For example, insecticide exposure is a key stressor affecting the population decline of pollinators, essential for ecosystem health and food production (3, 5).

A key obstacle to sustainable malaria control is the evolutionary arms race between mosquitoes and insecticide-based mosquito control. Strong selection pressures generated by insecticide-based agricultural pest and disease vector control activities have resulted in the independent evolution of a diverse range of mechanisms that confer insecticide resistance (IR) phenotypes in numerous insect species (6). One of the earliest described IR mechanisms was the emergence of *kdr*, mediated by mutations in the target site of pyrethroid and organochlorine insecticides, located in the voltage-gated sodium channel gene (*Vgsc*), which play key roles in the transmission of action potentials along neurons and are an essential component of the nervous system (7). These *kdr-*driven resistance phenotypes appeared rapidly after the introduction of the organochlorine dichloro-diphenyl-trichloroethane (DDT) spraying for insect control in the mid-20th century (8) and eventually evolved to confer resistance to pyrethroids (9, 10), the key ingredient in ITNs - the first line of defence against malaria. In an era of stalling gains in malaria control (2), and concerted efforts both to develop a new generation of ITN and IRS products (11, 12) and proactively manage the deployment of existing insecticides to maximise efficacy, intensified surveillance, including genomic surveillance (13, 14), of malaria vector populations is critical for providing real-time warning of insecticide resistance emergence.

Resistance to all major insecticide classes is common in *An. funestus* and is primarily mediated through the increased activity of enzymes that bind to and metabolise insecticides (metabolic resistance) (15, 16). This contrasts with another major vector *An. gambiae* where resistance is mostly conferred by a combination of metabolic and target-site resistance (6). In a previous study, we reported insecticide resistance phenotypes across Tanzania and found that, in the Morogoro region, resistance to DDT was present(17).

In this study, we report the findings of phenotypic and genomic surveillance done in Tanzania to understand the evolution and spread of insecticide resistance in *Anopheles funestus* - the dominant malaria vector in Eastern and Southern Africa (16). We report the first discovery of *kdr* mutations in *An. funestus*. We discover that, in Tanzanian *An. funestus*, *kdr* confers resistance to DDT, but not deltamethrin, despite a complete ban on DDT use for agriculture and vector control in Tanzania since 2008 by the Stockholm Convention (18). We suggest environmental contamination from extensive DDT stockpiles (19), or unofficial agricultural use, as possible causes. The emergence of *kdr*, which threatens the control of major crop pests and vectors of disease, such as *An. gambiae* and *Aedes aegypti* (20), highlights the potential of chemical insecticide contamination or unofficial use to exert unexpected and potentially harmful impacts on public health.

## Materials and methods

All scripts and Jupyter Notebooks used to analyse genotype and haplotype data, and produce figures and tables are available from the GitHub repository: https://github.com/tristanpwdennis/kdr_funestus_report_2023.

## Mosquito collection

**Fig. 1:**
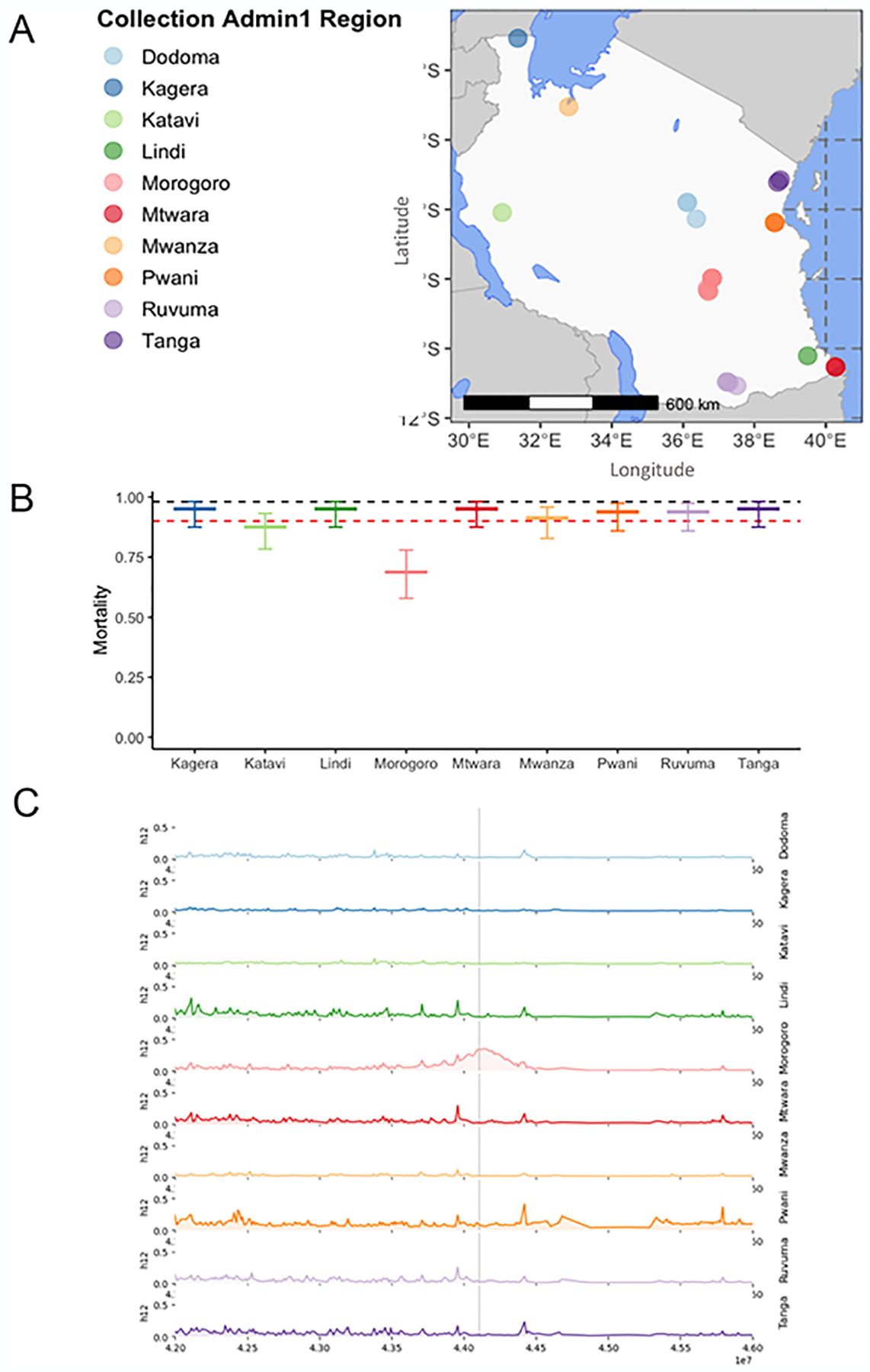
**(A)** Map of *An. funestus* collection locations. Points indicate sample collection locations. The point colour indicates the administrative region from which samples were collected. **(B)**: Phenotypic insecticide resistance profile of *An. funestus* to DDT using bioassay data adopted from our recent surveillance (17). The colours and the X-axis represent the various regions where the bioassays were conducted, and the error bars are 95% confidence interval. The black and red dotted lines on the Y-axis represent the 98 and 90% mortality thresholds. (**C**) **H12** selection scans of *An. funestus* chromosome 3RL, coloured and windowed by sample collection region (where n>20 – see Supp Table 2). The X-axis indicates the position (in base-pairs (bp)), and the Y-axis indicates the selection statistic H12. The Grey line indicates the location of the *Vgsc* gene. Note Mwanza region is absent from panel **C** as there were too few samples (n<20) to perform a selection scan.

*Anopheles funestus* samples analyzed in this study were collected from ten administrative regions in Tanzania: Dodoma, Kagera, Katavi, Lindi, Morogoro, Mtwara, Mwanza, Pwani, Ruvuma, and Tanga (Figure. 1A). The collections were done as part of a countrywide *Anopheles funestus* surveillance project in Tanzania and were subsequently incorporated into the MalariaGEN *Anopheles funestus* genomic surveillance project database (https://www.malariagen.net/projects/anopheles-funestus-genomic-surveillance-project). Mosquitoes were collected in households between 2017 and 2023 using CDC light traps and mechanical aspirators. They were sorted by sex and taxa and *An. funestus* group mosquitoes were preserved individually in 96-well plates containing 80% ethanol.

## Whole genome sequencing and analysis

The samples were processed as part of the *Anopheles funestus* genomics surveillance MalariaGEN Vector Observatory (VObs) project (https://www.malariagen.net/mosquito). Genomic DNA was extracted from individual mosquitoes using DNeasy Blood and Tissue Kits (Qiagen, Germany). A single band confirmed the DNA purity and integrity on 1% agarose gel and a minimum DNA concentration of 20 ng/μl on a Qubit^®^ 2.0 fluorometer. Samples that passed quality control, were individually whole-genome-sequenced commercially at 30X. The sequencing data have been deposited in the European Nucleotide Archive (https://www.ebi.ac.uk/ena/browser/home) under study number PRJEB2141.

Reads were aligned to the *An. funestus* reference genome AfunGA1 (21) with Burrows-Wheeler Aligner (BWA) version v0.7.15. Indel realignment was performed using Genome Analysis Toolkit (GATK) version 3.7-0 RealignerTargetCreator and IndelRealigner. Single nucleotide polymorphisms were called using GATK version 3.7-0 UnifiedGenotyper. Genotypes were called for each sample independently, in genotyping mode, given all possible alleles at all genomic sites where the reference base was not “N”. The aligned sequences in BAM format were stored in the European Nucleotide Archive (study number PRJEB2141).

The identification of high-quality SNPs and haplotypes was conducted using BWA version 0.7.15 and GATK version 3.7-0. Quality control involved the removal of samples with low mean coverage, removing cross-contaminated samples, running PCA to identify and remove population outliers, and sex confirmation by calling the sex of all samples based on the modal coverage ratio between the X chromosome and the autosomal chromosome arm 3R. Full quality control methods are available on the MalariaGEN vector data user guide (https://malariagen.github.io/vector-data/ag3/methods.html).

We used decision-tree filters that identify genomic sites where SNP calling and genotyping are likely to be less reliable. More information on site filters can be found in the MalariaGEN vector data user guide. Genotypes at biallelic SNPs that passed the decision-tree site filtering process were phased into haplotypes using a combination of read-backed and statistical phasing. Read-backed phasing was performed for each sample using WhatsHap version 1.0 [https://whatshap.readthedocs.io/]. Statistical phasing was then performed using SHAPEIT4 version 4.2.1 [https://odelaneau.github.io/shapeit4/].

Complete specifications of the haplotype phasing pipeline are available from the malariagen/pipelines GitHub repository (https://github.com/malariagen/pipelines/tree/master/pipelines/phasing-vector).

## Identification of SNPs on *Vgsc*

To identify the *An. funestus Vgsc* gene and the variant that confers target-site resistance we performed alignments between the *An. gambiae* VGSC transcript AGAP004707-RD in AgamP4.12 gene set from the Ag1000 phase 3 data resource (https://www.malariagen.net/data/ag1000g-phase3-snp) and AFUN2_008728 from the *An. funestus* AfunF1.3 dataset. We extracted single nucleotide polymorphism (SNPs) altering the amino acid of VGSC protein from the *An. funestus* dataset and computed the allele frequency on the mosquito cohorts defined by the region and year of collection (See Supp. Table 1 for per region/year sample numbers). Under selection pressure various alleles are expected to increase in frequency; we therefore filtered out variant alleles with a frequency lower than 5% resulting in a list of 8 variant alleles. Multiple sequence alignments of *An. funestus Vgsc* against *An. gambiae* and *M. domestica* were performed using MEGA v11.013 (22).

## Population genetic analyses

We searched for signatures of selective sweeps on the *Vgsc* gene using the *G123 and H12* selection statistics (23, 24). H12 selection scans were performed on *An. funestus* genotypes by collection region where sample *n*>20 (see Figure 1A and Supp Table 2) using the *h12_gwss* function in the malariagen_data python API (https://malariagen.github.io/malariagen-data-python/latest/Af1.html). Linkage disequilibrium (Rogers and Huff’s R-squared) (25) between the 8 *Vgsc* alleles was calculated using the *rogers_huff_r_between* in scikit-allel (https://zenodo.org/record/4759368). Haplotype clustering was performed by performing hierarchical clustering on a Hamming distance matrix, inferred from phased *An. funestus* haplotypes, using the Scipy library (https://scipy.org/citing-scipy/). Clustering dendrogram, and bar plot of amino acid substitutions, were plotted using the seaborn library (*48*).

## Association of L976F and P1842S alleles with insecticide resistance

Due to difficulties in finding *An. funestus* immature stages and unsuccessful attempts to get sufficient offspring from wild blood-fed females, we adopted a previously tested approach relying on unfed and non-gravid females of unknown ages for the resistance bioassays (26, 27). To test for associations between the identified mutations and IR, we exposed wild non-blood-fed *An. funestus* mosquitoes of unknown ages to standard doses of deltamethrin and DDT insecticides following the WHO tube assays. The bioassays were conducted as part of a countrywide insecticide resistance surveillance in Tanzania (17). For each insecticide, we randomly separated phenotypically resistant mosquitoes (i.e., alive 24 hours post-exposure) and susceptible (i.e., dead 24 hours post-exposure). For DDT we had 10 alive and 10 dead and for deltamethrin 29 alive and 27 dead. DNA was extracted from individual mosquitoes using DNeasy Blood and Tissue kit (Qiagen, Germany). The mosquitoes were identified at the species level using species-specific primers that can distinguish *An. funestus* from the other members of the group (28). To establish if the two *kdr* variants are associated with insecticide resistance, we designed PCR primers from *An. funestus Vgsc* (Gene ID: LOC125769886) to amplify domain IIS6 (L976F); kdrL976F_FWD 5’- TGTGCGGTGAATGGATCGAA-3’ & kdrL976F_REV 5’-CGCTTCAGCGATCTTGTTGG-3’, and C-terminal (P1842S) kdrP1842S-FWD 5’-CTACCCGGGAAATTGTGGCT-3’ & kdrP1842S_REV 5’-TGCCACCATCGTTTCCGTTA-3’. Each 20μl reaction volume contained 10μl GoTaq^®^ Green Master Mix (Promega, USA), 1μl (0.5μM final concentration) of forward/reverse primer, 1μl of DNA, and 7μl nuclease-free water. The thermocycler conditions were 94°C (5min), 30 cycles of 94°C (1min), 58°C (30sec), and 72°C (30sec), and a final extension of 72°C 10min. The DNA fragments were separated on a 1% agarose gel, cut, purified using PureLink^™^ Quick Gel Extraction Kit (Invitrogen), and commercially Sanger sequenced. Collectively, we sequenced 76 individual mosquitoes: 56 from deltamethrin and the rest from the DDT bioassays. The frequencies of the wild type and mutant alleles were determined and correlated with phenotypes using generalised linear models in R-software v4.1.1.

## Data analysis

To determine the phenotypic resistance to DDT and deltamethrin, we calculated the percentage mortality from the bioassays following WHO guidelines (29). Details of the modelling approach accounting for potential mortality variabilities due to extrinsic factors and the resistance profiles across Tanzania are outlined in Odero *et.al* (17). To explore the genetic association between L976F and P1842S alleles with DDT and deltamethrin resistance phenotype, we used Haploview version 4.1 statistical software (30). Linkage disequilibrium was established by D’ and R2 parameters. Genetic association between alleles or haplotypes and the resistance phenotypes were conducted on alleles with allele frequency > 0.05 and in Hardy-Weinberg equilibrium. A Chi-square test was performed, and P-values were calculated for the allelic and haplotype frequencies in the alive and dead mosquitoes. The odds ratio for statistically associated and marginally significant alleles was calculated by comparing the related alleles with the rest.

## Results

As part of an insecticide resistance surveillance study in 10 sites across Tanzania (17) (Fig. 1A), we investigated phenotypic resistance (as measured by mosquito survival 24 hours following insecticide exposure) in *An. funestus* to the discriminating doses of DDT, deltamethrin (type II pyrethroid), or deltamethrin together with the piperonyl butoxide (PBO) synergist, which is increasingly used on ITNs (23) to restore susceptibility in pyrethroid-resistant populations in Tanzania. The mosquitoes were phenotypically resistant to deltamethrin in all Tanzanian regions, but PBO ubiquitously restored susceptibility (17). Unexpectedly, resistance to DDT was recorded in the Morogoro region (68%, CI 57.8 – 77.9), but resistance in other locations is also a possibility (17) (Fig. 1B).

To understand the genetic bases of resistance, we analysed whole-genome-sequencing (WGS) data from 333 mosquitoes sampled from 10 sites across Tanzania (Fig. 1A). We performed genome-wide selection scans (GWSS) with the H12 and G123 statistics (Figures S1 and S2) to test for evidence of selective sweeps in the *An. funestus* genome associated with known or novel IR loci (Fig. 1C; grouping samples by administrative region, including those collected at different time points (see Supp. Table 1 for per-group sample numbers). We detected a signal of elevated H12, indicating a possible selective sweep in the region containing the *Vgsc* gene (Chromosome 3, positions 44105643–44105644) in samples from the Morogoro region in the southeastern part of the country (Fig. 1C). Notably, *Vgsc* encodes for the voltage-gated sodium channel, where DDT binds in mosquitoes, and where mutations are strongly linked to resistance in *An. gambiae* (11). In Kagera, Katavi, and Mwanza regions, there was no visible sign of a selective sweep at or near the *Vgsc* region. In Dodoma, Lindi, Ruvuma, and Tanga, there were peaks of elevated H12 near *Vgsc*, but these appeared within the context of relatively high H12 across the chromosome (Fig. 1C, S1 and S2).

**Fig. 2:**
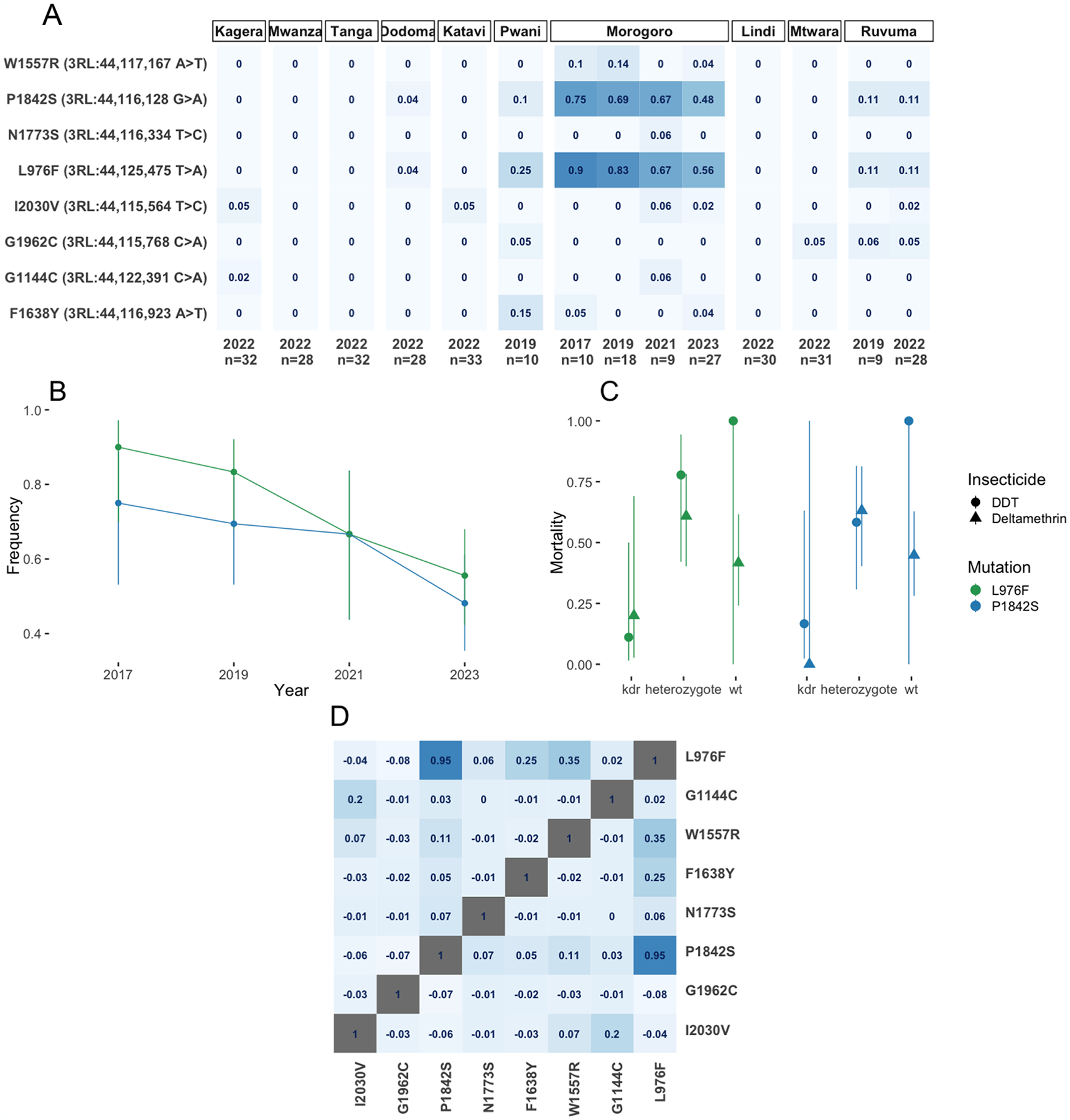
**(A)** Heatmap of *Vgsc* allele frequencies. Y-axis labels indicate mutation effect, chromosome position, and nucleotide change. X-axis labels indicate the collection date and heatmap intensity indicates frequency where darker = higher, with frequency labelled in each heatmap facet. The heatmap is panelled by the sample collection region. (**B)** L976F and 1842S frequencies, in the Morogoro region, over time. The y-axis indicates allele frequency, X-axis indicates the date. Line and point colour refer to mutation, specified in the legend. Bars indicate 95% confidence intervals. (**C**) Denotes the association of L976F and P1842S with resistance to Deltamethrin and DDT. Colour and panelling are by mutation, the x-axis indicates genotype, the y-axis indicates mortality, the point shape indicates the mean for each insecticide and the line indicates the 95% CI based on generalised mixed model prediction. (**D)** Heatmap of linkage disequilibrium (LD) (Rogers and Huff R) between nonsynonymous variants in the *Vgsc* gene at frequency > 5%. LD is indicated by fill colour. SNP effects and positions are labelled on the X and Y axes.

We searched our data for mutations in the *Vgsc* gene and found 8 amino acid substitutions occurring at frequencies greater than 5% (Fig. 2A). Of these, two alleles, L976F and P1842S occurred at the highest frequency (Fig. 2A). The frequencies of P1824S and L976F were highest in samples collected from Morogoro in 2017 (0.75 and 0.90 respectively) (Fig. 2A) and declined yearly, reaching their lowest frequency in samples collected in 2023 (0.48 and 0.56 respectively; *χ*^2^ = 12.15, p=0.0005; Fig. 2B). These mutations occurred at very low frequencies or were absent in all other locations (Fig. 2A). To understand their function, we aligned the *An. funestus Vgsc* sequence (Gene ID: AFUN2_008728.R15290) with that of *Musca domestica* (Gene ID: X96668) and *An. gambiae* (AGAP004707-RD AgamP4.12 gene set) (31). We found that the amino acid change at *An. funestus* L976F corresponded to L1014F in *M. domestica* and L995F in *An. gambiae* in domain II subunit 6 (IIS6) of the *Vgsc* gene (Table 1), which in *An. gambiae* species complex drastically increases IR to DDT and pyrethroids (10, 32). The second variant P1842S corresponded to P1874S in *An. gambiae* and P1879 in *M. domestica* and were all in the C-terminal domain (Table 1).

To explore the possible association between L976F and P1842S alleles with DDT and deltamethrin resistance, we genotyped surviving (resistant) and dead (susceptible) mosquitoes from IR bioassays for both L976Fand P1842S loci). Neither locus was associated with deltamethrin resistance: L976F (*χ*^2^ = 0.04, p = 0.84) and P1842S (*χ*^2^ = 0.59, p = 0.44). (Fig. 2C). We found a strong association with DDT resistance in mosquitoes carrying L976F (*χ*^2^ = 9.23, odds ratio = 11.0, p = 0.0024) and a marginally non-significant positive association for P1842S (*χ*^2^ = 3.75, p = 0.0528) (Fig. 2C).

**Fig. 3:**
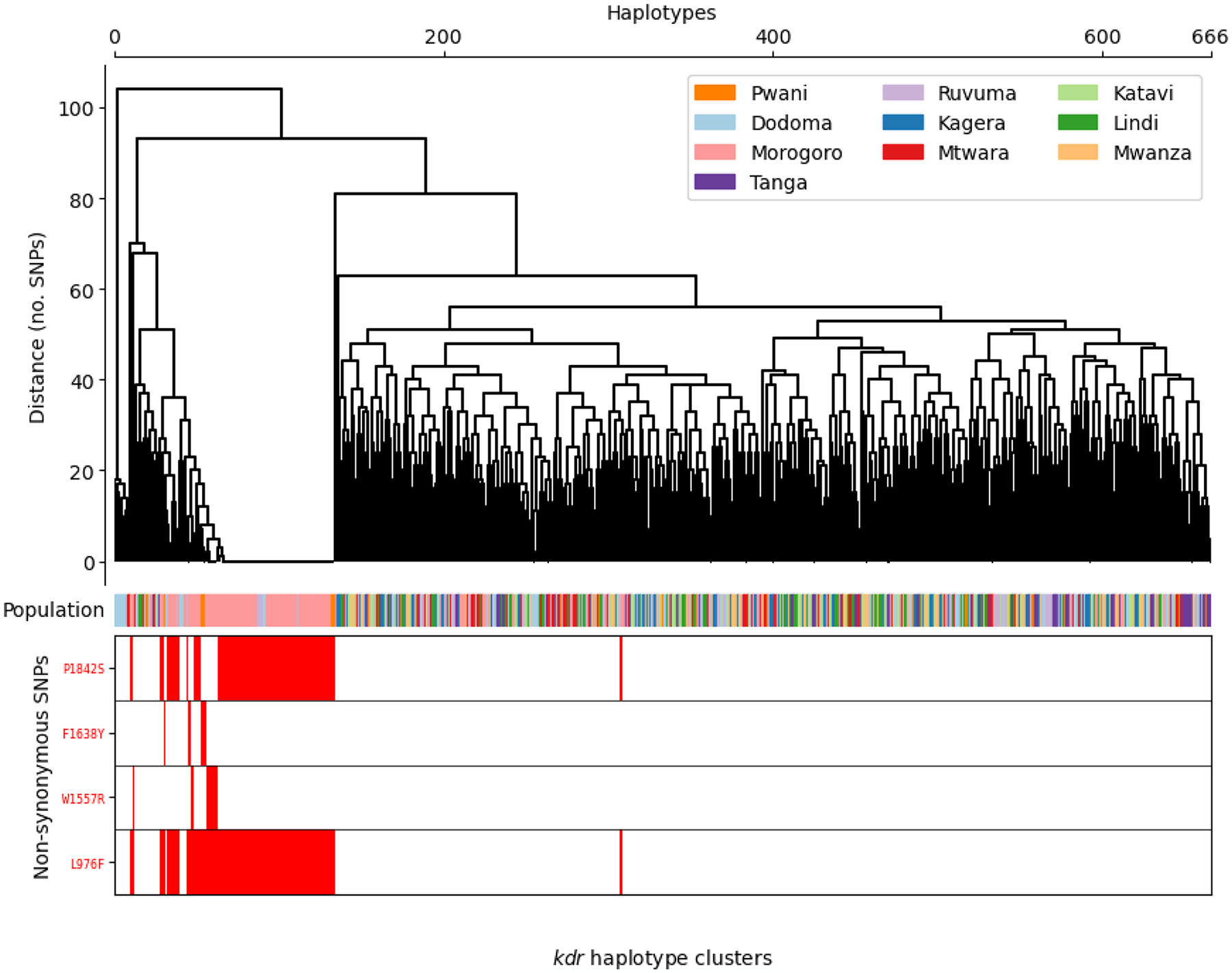
Clustering of haplotypes at the *Vgsc* gene (LOC125769886, 3RL:44105643–44156624). The dendrogram branch length corresponds to no. SNPs difference (y-axis). Tips correspond to individual haplotypes (x-axis). The coloured Population bar denotes the administrative region of origin (as described by the legend). Red blocks at the bottom indicate the presence of the labelled non-synonymous SNPs in the *Vgsc* gene.

To elucidate *Vgsc* haplotype structure in *An. funestus*, we computed pairwise linkage disequilibrium (LD) using the Rogers and Huff method (briefly, derived from r, the correlation across unphased genotypic values) (25), between nonsynonymous variants occurring at a frequency of > 5% in Tanzanian *An. funestus* (Fig. 2D). We found that P1824S occurred in tight LD with L976F (*D’*=0.95) (Fig. 2D). Of other non-synonymous polymorphisms, F1638Y and W1557R exhibited only weak LD with L976F (Fig. 2D). We constructed a haplotype clustering dendrogram from haplotypes in all 333 individuals, from the *Vgsc* gene (Fig. 3). The clustering dendrogram disclosed three major clades and three main combinations of the four most prevalent *Vgsc* alleles (Fig. 3). The most striking signal was a subclade of identical, or near-identical haplotypes containing both L976F and P1842S (Fig. 3), indicating a selective sweep on a combined L976F/P1842S haplotype. This combined haplotype was present at higher frequencies in the Morogoro region relative to the neighbouring regions of Pwani, Ruvuma, and Dodoma (Fig. 3). Most amino acid substitutions were present in a single clade in samples from Pwani, Dodoma, Ruvuma, and especially Morogoro (Fig. 3).

## Discussion

In a genomic surveillance study in Tanzanian *An. funestus*, we discovered eight novel *Vgsc* mutations. L976F, confers *knockdown resistance* (*kdr*), occurring in tight linkage disequilibrium with, P1842S, and at high frequencies (up to 90%) in the Morogoro region over 4 years, with limited spread to neighbouring regions. The mutation L976F showed an association with resistance to DDT, but not to pyrethroid insecticides. The role of *kdr* in pyrethroid resistance phenotypes in other *Aedes, Culex* and *Anopheles* vectors, makes the discovery of *kdr* in *An. funestus* a significant and unwelcome development that has the potential to pose a new threat to vector control in the region. Reassuringly, a lack of association between *kdr* and deltamethrin resistance indicates that the emergence of *kdr* is not linked to, nor is presently likely to threaten, the mass rollout of PBO-pyrethroid bed nets currently underway in Tanzania as a response to IR (34). The emergence of *kdr* resistance to DDT suggests that future use of DDT for IRS may become even less favoured. However, this does not preclude a role for *kdr* in the *An. funestus* IR armamentarium in the future and an urgent follow-up study is required to monitor the evolution of vector DDT resistance and determine whether *kdr confer* resistance phenotypes to other widely used pyrethroids, such as permethrin, and alpha-cypermethrin, as well as other insecticide families, especially PBO and pyrrole formulations currently being rolled out in new ITN products across the African continent (35). The lack of association of *kdr* with pyrethroid resistance might be due to the strong metabolic resistance shown to pyrethroids in *An. funestus*, reducing the benefit of *kdr* (15). The association of *kdr* with resistance to DDT but not pyrethroids, combined with selection signals and recently declining *kdr* allele frequencies where we have time series, suggests recent-past, rather than contemporary selection, perhaps due to factors other than the current use of public health pesticides.

This discovery raises intriguing questions about the conditions that have enabled the emergence of *kdr* in *An. funestus*. Our data suggests that *Vgsc* mutation in *An. funestus* do not confer target-site resistance to pyrethroids, indicating a possible explanation as to why, despite extreme selection pressures imposed by pyrethroid control have facilitated widespread propagation of resistant *Vgsc* haplotypes across the African continent in *An. gambiae* (31), the emergence of *kdr* in Tanzanian *An. funestus* remains relatively localised. Mechanistic studies, including expression studies of mutant *Vgsc* proteins in *Xenopus* oocytes (36), will enable comparisons between taxa that will elucidate this further.

**Fig. 4:**
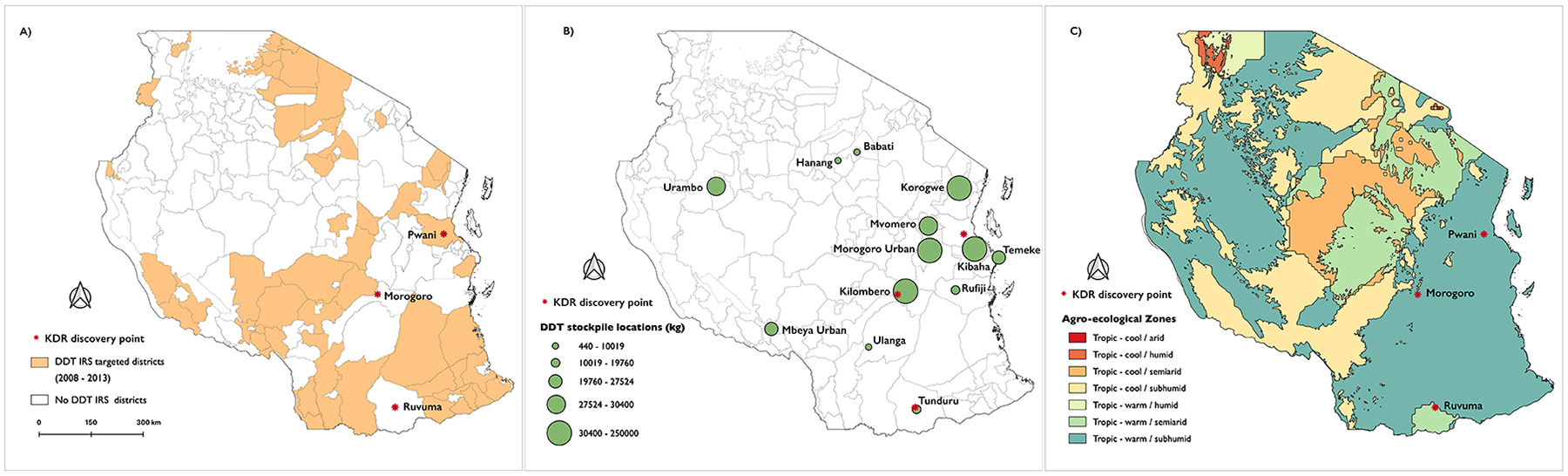
(**A**) Tanzanian National Malaria Control Programme (NMCP) indoor residual spraying strategy 2008 – 2012. The colours indicate districts where DDT spraying was planned. (**B**) DDT stockpile locations with the size of the circle indicating the stockpile quantities. (**C**) Agro-ecological zones in Tanzania with colours on the map denoting the categories indicated in the figure key.

If the ubiquitous use of pyrethroids in vector control did not select for the emergence of *kdr*, whence *kdr?* Even more curiously, the apparent decline of *kdr* allele frequencies between 2017 and 2023 suggests that the selection pressure causing the emergence of *kdr* has eased (although non-uniform sample sizes per time-point make confident assertion of this difficult). DDT is a largely obsolete, banned, pesticide that is no longer widely used for vector control in Tanzania, or in Africa as a whole, due to its bio-accumulative and toxic properties - with the most well-known effects being egg-shell thinning properties among birds (37), and associations with human cancers (38). There is no record of DDT use in the last decade for agriculture or vector control in the Morogoro region, or Tanzania as a whole, where the production, importation, and usage of DDT have been banned since 2009 (39), except for limited use in malaria vector control. In 2008, Tanzania rolled out an ambitious malaria vector control strategy relying on large-scale use of DDT for indoor residual spraying (IRS), implemented in 60 districts across the country (Fig. 4A), and discontinued in 2010 (40). Morogoro, where we detected *kdr*, was not part of this expanded campaign. Before the ban, Tanzania imported large stockpiles of DDT mostly for agricultural pest control (Fig. 4B). Following the ban, there have been anecdotal reports of continued illegal use of DDT amongst farmers to date (41). The Africa Stockpiles Programme (ASP) was launched in 2005 to eliminate stockpiles of obsolete pesticides, including DDT. At this time, it was estimated that Tanzania still possessed approx. 1,500 tonnes of obsolete pesticides (42), including a DDT stockpile of 30 tons (as of 2012) (19), approximately 50 km away from where DDT-resistant *An. funestus* were detected in this study, and 156 tons were in Morogoro town (19) (Fig. 4B). The ASP and the Tanzanian Government eliminated 100% of inventoried publicly held DDT stockpiles and conducted extensive environmental remediation by programme closed in 2013 (43). However, extensive DDT contamination remains (44), and DDT remains in widespread use by private individuals (41). The coincident proximal location of high levels of *kdr* in *An. funestus* with large past DDT stockpiles as well as the presence of widespread DDT contamination and private usage leads us to hypothesise that the two most likely scenarios of *kdr* emergence in *An. funestus* are contamination of local larval breeding sites from agricultural or stockpiled DDT (Fig. 4B, C), and further investigation is needed to establish why *kdr* did not emerge and sweep through the population during periods of widespread DDT usage in the 20th century The removal of DDT stockpiles by the ASP, and ongoing environmental remediation, may have contributed to reduced selection pressure on *kdr*, evident from declining frequency in Morogoro (although small and uneven sample sizes make confident assertion of this trend difficult). Continued monitoring of allele frequencies and future studies of *kdr* frequencies targeted towards sites of known DDT contamination will establish whether this hypothesis is correct.

In *Silent Spring* (1962), Rachel Carson brought for the first time into the public eye the unpredictable and often remote impacts of anti-insect chemical agents on human health and nature “On one hand delicate and destructible, on the other miraculously tough and resilient, and capable of striking back in unexpected ways” (45). Further study of *kdr* in *An. funestus* will enable the identification of the origin of this mutation and make clear the full implications of its presence in the population for vector control. Whether the emergence of *kdr* in *An. funestus* is caused by vector control, unlicensed DDT usage in agriculture, or exposure to stockpiled DDT, our findings underscore the legacy of *Silent Spring* by reinforcing the potential for pesticides and organic pollutants to exert inadvertent influences on animal biology that may have profound and unfortunate consequences for public health.

## Supplementary Material

Supinfo1 **Figure S1.** G123 selection scans of *An. funestus* chromosome 3RL, coloured and windowed by sample collection region (where n>20 – see Supp Table 2). X-axis indicates the position (in base-pairs (bp)), Y-axis indicates the selection statistic G123. The Grey dotted line indicates the location of the *Vgsc* gene. Note Mwanza region is absent as there were too few samples (n<20) to perform a selection scan.

Supinfo2 **Figure S2.** H12 selection scans of *An. funestus* chromosome 3RL, coloured and windowed by sample collection region (where n>20 – see Supp Table 2). The X-axis indicates the position (in base-pairs (bp)), and the Y-axis indicates the selection statistic H12. The Grey dotted line indicates the location of the *Vgsc* gene. Note Mwanza region is absent as there were too few samples (n<20) to perform a selection scan.

Supinfo3

Supinfo4

## Figures and Tables

**Table 1:** Comparative non-synonymous nucleotide variation in the voltage-gated sodium channel gene. The position is relative to the *Anopheles funestus* strain FUMOZ reference, chromosome arm 3RL. Codon numbering according to *Anopheles funestus Vgsc* transcript AFUN2_008728.R15290, *Anopheles gambiae* transcript AGAP004707-RD in gene set AgamP4.12, and *Musca domestica* EMBL accession X96668 Williamson *et al*. (33).

Position	*An. funestus*	*An. gambiae*	*M. domestica*	Domain
3RL:44,115,564 T>C	I2030V	I2061	P2063	COOH
3RL:44,115,768 C>A	G1962C	A1994	P1997	COOH
3RL:44,116,128 G>A	P1842S	P1874S	P1879	COOH
3RL:44,116,334 T>C	N1773S	N1805	N1810	
3RL:44,116,923 A>T	F1638Y	F1670	V1675	
3RL:44,117,167 A>T	W1557R	W1589	W1594	
3RL:44,122,391 C>A	G1144C	G1173	G1180	
3RL:44,125,475 T>A	L976F	L995F	L1014	IIS6

## Data Availability

The whole genome sequencing data generated in this study have been deposited in the European Nucleotide Archive (https://www.ebi.ac.uk/ena/browser/home) under study number PRJEB2141. The partial Sanger sequence data for L976F and P1842S are available through Github https://github.com/tristanpwdennis/kdr_funestus_report_2023/blob/main/data/kdr_sequenced_sanger.fasta.
