# Supplementary figures and images for: Discovery of knock-down resistance in the major African malaria vector Anopheles funestus
Source: Mol Ecol. Author manuscript; Available in PMC 2024 Nov 6. (PMC11537839; doi:10.1111/mec.17542)

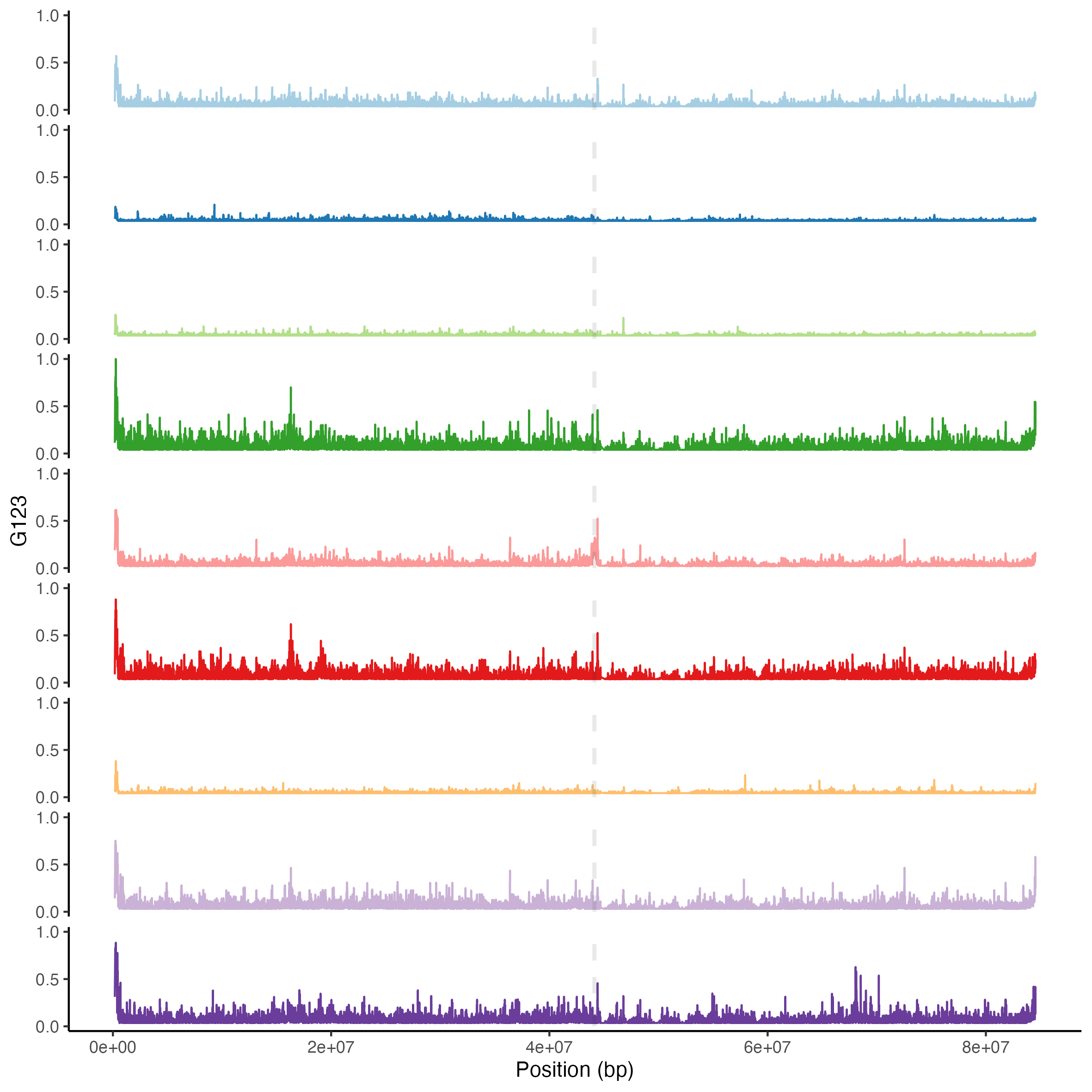

Supplement: Supinfo1 — Figure S1. G123 selection scans of An. funestus chromosome 3RL, coloured and windowed by sample collection region (where n>20 – see Supp Table 2). X-axis indicates the position (in base-pairs (bp)), Y-axis indicates the selection statistic G123. The Grey dotted line indicates the location of the Vgsc gene. Note Mwanza region is absent as there were too few samples (n<20) to perform a selection scan. [file NIHMS2025771-supplement-Supinfo1.tiff]

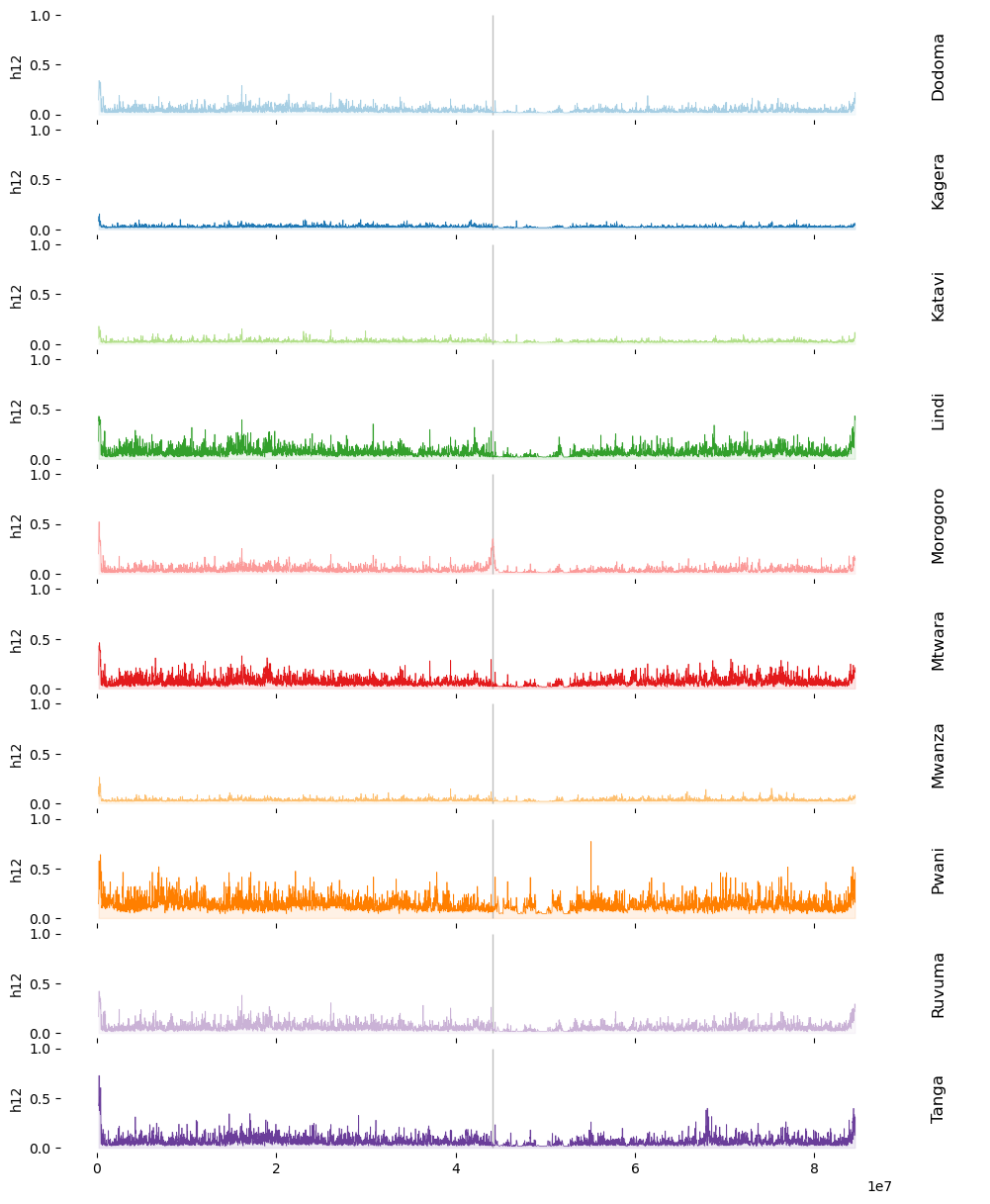

Supplement: Supinfo2 — Figure S2. H12 selection scans of An. funestus chromosome 3RL, coloured and windowed by sample collection region (where n>20 – see Supp Table 2). The X-axis indicates the position (in base-pairs (bp)), and the Y-axis indicates the selection statistic H12. The Grey dotted line indicates the location of the Vgsc gene. Note Mwanza region is absent as there were too few samples (n<20) to perform a selection scan. [file NIHMS2025771-supplement-Supinfo2.png]
